# Supplementary material for: Complexome profiling of the Chlamydomonas psb28 mutant reveals TEF5 as an early PSII assembly factor
Source: Plant Cell. 2025 Mar 17;37(6):koaf055. doi: 10.1093/plcell/koaf055 (PMC12164590; doi:10.1093/plcell/koaf055)
Supplement: koaf055_Supplementary_Data [file koaf055_supplementary_data.zip › psb28_interactive.zip!/plots/intensity/qualityPlots/peptides/_Cre10.g439000.t1.1.html]

### Cre10.g439000.t1.1

Trivial name:   
SeqCoverage [median]: 7.50  
Mass: 83.07 kDa  
  
 

| Sequence | MissedCleavage | Mass [Da] | wtIntensity | muIntensity | Score |
| --- | --- | --- | --- | --- | --- |
| DLTVATPR | 0 | 871.48 | 895.22 | 0.00 | 91.90 |
| LGEFDEAVVSR | 0 | 1220.60 | 0.00 | 834.00 | 59.43 |
| LLAEYLSER | 0 | 1092.58 | 342.71 | 0.00 | 70.92 |
| LQAAVENYMGLITASR | 0 | 1735.89 | 0.00 | 498.61 | 63.76 |
| VLAQSGISYVSVGHR | 0 | 1571.84 | 0.00 | 483.12 | 35.66 |
|  |  |  | 1237.9 | 1815.7 |  |
